# Quality of life is impaired in shrimp allergic adults and caregivers

**DOI:** 10.3389/falgy.2025.1622538

**Published:** 2025-12-08

**Authors:** Melissa L. Hearrell, Sara Anvari, Sharon Chinthrajah, Sana Hasan, David P. Huston, Evan Li, Chen-Hsing Lin, Edwin Kim, Andreas L. Lopata, Sarbjit Singh Saini, Sayantani Sindher, Panida Sriaroon, Bin Su, Julie Wang, Carla M. Davis

**Affiliations:** 1 Division of Immunology, Allergy, & Retrovirology, Department of Pediatrics, Baylor College of Medicine, Houston, TX, United States; 2 Sean N Parker Center for Allergy and Asthma Research, Stanford University, Stanford, CA, United States; 3 Division of Immunology, Allergy, and Rheumatology, Department of Medicine, Baylor College of Medicine, Houston, TX, United States; 4 Division of Allergy & Immunology, Houston Methodist Immunology Center, Houston Methodist Hospital, and Texas A&M School of Medicine, Houston, TX, United States; 5 Division of Rheumatology, Allergy, and Immunology, The University of North Carolina at Chapel Hill School of Medicine, Chapel Hill, NC, United States; 6 Australian Institute of Tropical Health & Medicine and Tropical Futures Institute-Singapore, James Cook University, Townsville, QLD, Australia; 7 Department of Medicine, Division of Allergy and Immunology, Johns Hopkins University School of Medicine, Baltimore, MD, United States; 8 Department of Pediatrics, Division of Allergy and Immunology, University of South Florida Morsani College of Medicine, Tampa, FL, United States; 9 Department of Molecular Virology & Microbiology, Baylor College of Medicine, Houston, TX, United States; 10 Division of Pediatric Allergy and Immunology, Icahn School of Medicine at Mount Sinai, Jaffe Food Allergy Institute, New York, NY, United States; 11 Department of Pediatrics and Child Health, Howard University College of Medicine, Washington DC, United States

**Keywords:** shrimp, shrimp allergy, quality of life, seafood, food allergy, food allergy (FA), survey, anaphylaxis

## Abstract

**Rationale:**

Shrimp (*Litopenaeus vannamei*) allergies (SA) can result in allergic reactions ranging from life threatening severe anaphylaxis to oral allergy syndrome. SA may pose lifestyle restrictions on daily life and interfere with social relationships and school performance, but this has not been thoroughly investigated. We examined the QoL in SA adults and caregivers.

**Methods:**

A QoL online questionnaire adapted from the validated Food Allergy Quality of Life Questionnaire (FAQLQ) was administered between September 30, 2023–July 15, 2024 to adults and caregivers of at least one SA child. Descriptive statistics, Wilcoxon rank sum test, and Fisher exact test were used to determine QoL and desire for treatment in SA subjects. Comparisons were made between SA adults with and without children with SA.

**Results:**

Eighty-six participants completed the survey. Sixty-four (74%) SA adults did not have SA children, and 6 (7%) were SA adults with SA children. Eighty-one percent of SA adults found SA at least moderately to extremely troublesome, and 83% felt other people underestimated problems caused by SA. Seventy percent of SA adults were interested/very interested in a treatment and, of those interested, 47% wanted treatment to enable eating a serving size of shrimp. The small cohort of SA adults with a SA child may have been more likely to have concerns about allergic reaction compared to SA adults without a SA child. [OR, 4.4 (CI, 1–21)]

**Conclusions:**

SA adults report impaired QoL and a desire for treatment to eat a serving size of shrimp. The majority of SA people have impaired QoL.

## Introduction

Shrimp allergy (SA) is a form of shellfish allergy that is one of the leading causes of severe allergic reactions (1–3). It can result in reactions ranging from life threatening severe anaphylaxis to oral allergy syndrome. Shellfish allergies are a global problem, and one of the most common food allergies in the United States (US), especially among shoreline counties where consumption is high. US SA accounts for approximately 25% of adult food allergies and 20% of pediatric food allergies, representing approximately 3% of the adult population and 1.3% of the pediatric population (1, 2). SA is considered a lifelong allergy with a low rate of spontaneous resolution (4–6). Even though SA has a high prevalence, causes serious reactions, and rarely resolves naturally, the impact of SA on quality of life (QoL) has not been thoroughly studied (6, 7).

Shellfish allergy has been documented to increase patient psychosocial burden. Food allergies can result in poor QoL that can manifest as anxiety and withdrawal from social situations and social anxiety. SA may pose lifestyle restrictions on daily life and interfere with social relationships and school performance, but this has not been thoroughly investigated. This study was initiated to assess the impact of SA on QoL of patients with SA and caregivers of children with SA and to determine the need or desire for adult and pediatric treatment options for SA.

## Methods

This was a cross sectional study where adults with SA and/or caregivers of at least one SA child were asked to take a comprehensive QoL online questionnaire adapted from the validated Food Allergy Quality of Life Questionnaire (Supplementary Appendix A), a disease specific self-administered instrument which includes measures relevant to family activities, social activities, school, food preparation, health concerns, and emotional issues (8, 9). The questionnaire was distributed through a QR code on a flyer that was posted in the investigators' allergy/immunology clinics and through email from the following patient advocacy groups: Texas Children's Hospital Food Allergy Network, CURED Foundation, Food Allergy Research Education. The questionnaire allowed caregivers of SA children (age 0–17 years) to respond as proxies. The questionnaire was adapted by adding 4 questions pertaining to shrimp allergy treatment (See Supplementary Appendix A). The questionnaire was administered between September 30, 2023–July 15, 2024. Survey responses were collected, and descriptive statistics were summarized. Comparisons were made and evaluated between three patient groups. One group was categorized as SA adults with no SA children, the second group was SA adults with at least one SA child, and a third group was adults without SA that have at least one SA child. The Wilcoxon rank-sum test and Fisher exact test were used for analysis with a statistical significance level set at 0.05 to determine the QoL and desire for treatment among SA patients and adult caregivers with at least one SA child.

## Results

Eighty-six participants completed the survey. Sixty-four (74%) were SA adults without a SA child, 6 (7%) were SA adults with one SA child, and sixteen (19%) were adults without SA with at least one SA child. From the 22 respondents with at least one SA child, there were twelve (55%) respondents that had a SA child between ages 0 and 12, 5 (23%) respondents that had a SA child between 13 and 17 years of age, and 5 (23%) respondents that had both at least one SA child in the 0–12 age group and at least one SA child in the 13–17 age group.

**Figure 1:**
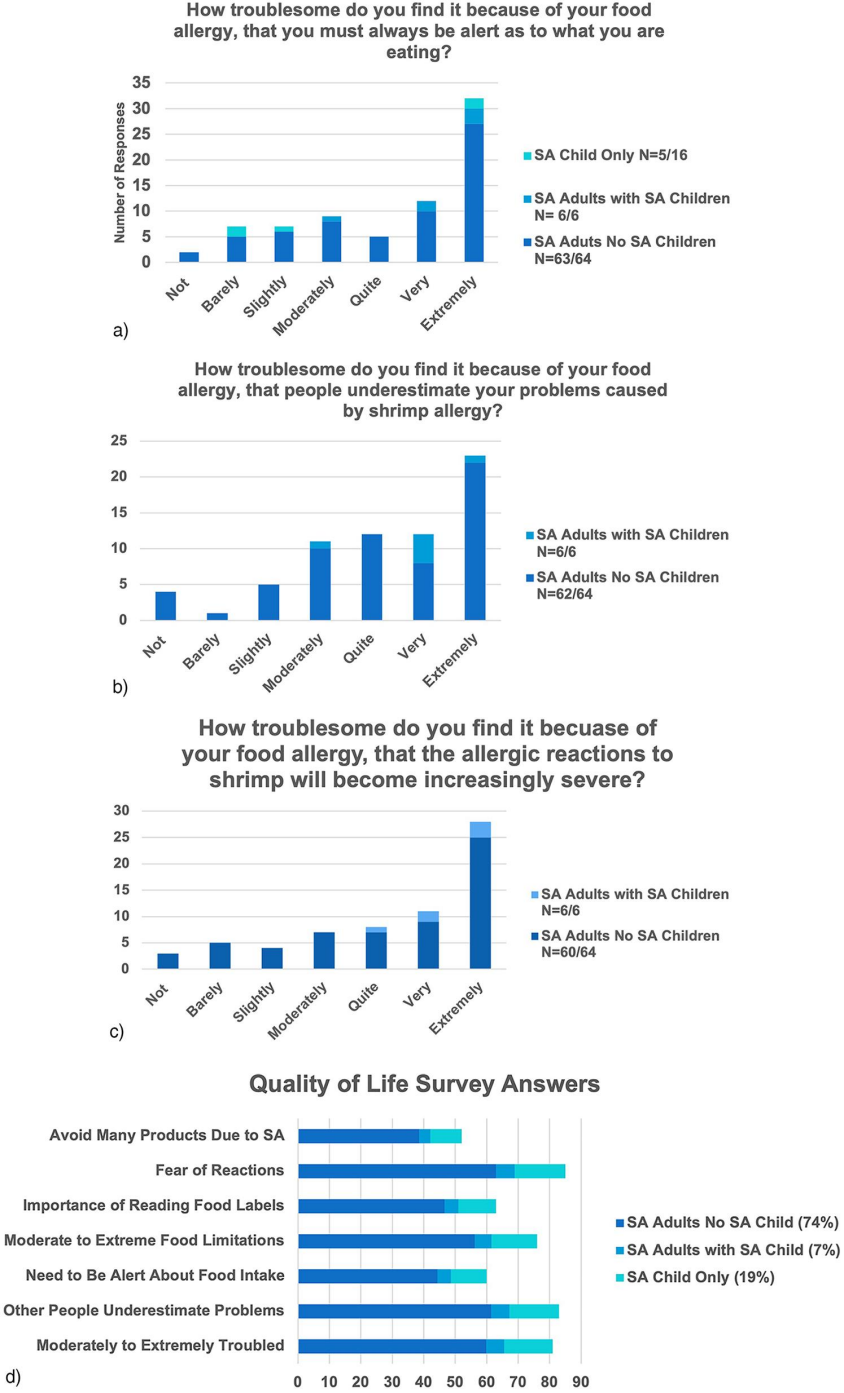
**(a)** Answer from Shrimp Allergic (SA) Adults to the question, “How troublesome do you find it, because of your food allergy, that you much always be alert as to what you are eating?” For each category (SA Child Only, SA Adults with SA Child, SA Adults No SA Child), the number of participants from the cohort that answered this question in the survey is given after the category label. **(b)** Answer from Shrimp Allergic (SA) Adults to the question, “How troublesome do you find it, because of your food allergy, that people underestimate your problems caused by shrimp allergy?” For each category (SA Child Only, SA Adults with SA Child, SA Adults No SA Child), the number of participants from the cohort that answered this question in the survey is given after the category label. **(c)** Answer from Shrimp Allergic (SA) Adults to the question, “How troublesome do you find it, because of your food allergy, that allergic reactions will become increasingly severe?” For each category (SA Child Only, SA Adults with SA Child, SA Adults No SA Child), the number of participants from the cohort that answered this question in the survey is given after the category label. **(d)** Quality of Life Survey Percent Positive Answers from Shrimp Allergic (SA) Adults. Percentage of the cohort in each category is listed after the category (SA Child Only, SA Adults with SA Child, SA Adults No SA Child).

Eighty-one percent of SA adults found SA at least moderately to extremely troublesome, and 83% felt other people underestimated problems caused by SA (Figures 1a–c). When participants were divided into groups with the first group being SA adults with no SA children, the second group being SA adults with at least one SA child, and the third group being adults without SA that have at least one SA child, there was very little statistical difference in the QoL measures between the different groups. SA adults with a SA child were more likely to have concerns about dealing with an allergic reaction compared to SA adults without a SA child. However, this small subgroup (*n* = 6) of SA adults with SA children has limited power and interpretability. Otherwise, the groups had common QoL concerns regarding their SA.

Eighty-two percent at least moderately feared SA reactions of increasing severity. Eighty-five percent indicated they were at least moderately scared of an allergic reaction with 55% reporting that they were very or extremely frightened of an allergic reaction (Figure 1c). Seventy percent of SA adults were interested/very interested in a treatment and, of those interested, 47% wanted treatment to enable eating a serving size of shrimp. All respondents with an adolescent child with SA were interested in a treatment for SA and 78% of parents with a SA child 12 years of age or under were interested in a treatment for SA.

Among the total population, 60% indicated a constant need to be very or extremely alert as to what they were eating. Seventy-six percent reported at least moderate limitations on food availability with 44% indicating foods were very or extremely limited. Sixty-three percent indicated that it was very important or extremely important to read food labels. Eighty-nine percent reported some level of refusing of treats at school or work. Eighty-three percent were at least moderately troubled that people underestimate their allergy. Sixty-eight percent reported avoiding some products because of SA and 52% indicated that they avoid many products because of SA (Figure 1d).

## Discussion

The strengths of this study include the national US distribution of the survey, the focus on SA, the use of a validated survey instrument, and the novel findings of the experiences of SA impacting quality of life. This survey data and analysis indicate the majority of survey participants that have SA or are the caregiver of a child with SA have decreased QoL measures as a result of their SA. These decreased QoL measures range from hypervigilance about the foods they/their child eat to exclusion or isolation at work or school. The hypervigilance with foods and isolation at work or school could lead to school and work absenteeism or home schooling on days where these foods may be served in the work or school setting.

The limitations of this study include the cross sectional nature, the addition of non-validated questions about shrimp allergy, and lack of data regarding the response rate. Since the questionnaire allowed caregivers to serve as proxies for the children, proxy reporting may not fully reflect the child's experience, particularly for emotional and social domains. We also did not assess the demographics of the respondents so the generalizability of the responses is limited. Finally, the small subgroup of SA adults with SA children may limit the interpretability, overstate true findings and needs more exploration. Even with the above limitations, given the paucity of information regarding SA QoL in the literature, this study adds to our understanding of the impact of SA.

Most participants in this study report a desire for SA treatment and half of SA adults report a desire for treatment to facilitate eating a serving size of shrimp. Having a child with SA heightens some of these concerns, with SA caregivers experiencing greater concerns about treating allergic reactions. Preventative treatments like biological treatments, immunotherapy, or epitope vaccines would be helpful in the future and more studies to mitigate the impact of SA on QoL are needed.

## Data Availability

The raw data supporting the conclusions of this article will be made available by the authors, upon reasonable request.
