# Supplementary material for: Quality of life is impaired in shrimp allergic adults and caregivers
Source: Front Allergy. 2025 Dec 8;6:1622538. doi: 10.3389/falgy.2025.1622538 (PMC12719276; doi:10.3389/falgy.2025.1622538)
Supplement: Supplementary file 1 [file Datasheet1.pdf]

Quality of Life (QOL) ASSESSMENT FOR SPECIFIC FOOD ALLERGIES Dear Potential Research Subject:Food allergies have a significant impact on the quality of life of patients and their families, however, the quality of life of patients with several food allergies has not been well defined. We in the Immunology, Allergy, and Retrovirology Department at Baylor College of Medicine/Texas Children's Hospital would like to evaluate the effect on quality of life of patients and their caregivers who suffer from shrimp allergies. We are contacting you because you have been diagnosed with Food Allergies.The study will determine the quality of life for specific food allergens and takes approximately 5-10 minutes to complete. Please tick one box for each question and ensure that all questions are answered. If you fill out this anonymous questionnaire (survey), you are consenting or agreeing to take part in this research.You decide whether you want to take part of not. If you do not take part, you will lose none of your rights. It will not affect you badly in any way. You may decide to stop taking part at any time. Again, if you decide not to take part, it will not affect your rights or benefits. It will not change the health care you receive now or in the future. By deciding to take part you also note that your child understands and agrees to take part in this study according to his or her understanding.It will not cost you to take part in this study. We will not pay you to take part.If you have any questions about this survey or the study, please contact Dr. Carla M. Davis at 832-824-1319. If you have additional questions about your rights as a research subject, contact the Institutional Review Board for Human Subject Research for Baylor College of Medicine & Affiliated Hospitals at (713) 798-6970. Thank you for your time. Sincerely,

---

Are you at least 18 years old and personally have a shrimp allergy?

○ Yes
○ No

Food Allergy Quality of Life Questionnaire - Adult Form (≥18 years)

Please answer the following questions related to your shrimp allergy:

How troublesome do you find it, because of your shrimp allergy, that you...

	Not	Barely	Slightly	Moderately	Quite	Very	Extremely
Must always be alert as to what you are eating?	○	○	○	○	○	○	○
Are able to eat fewer products?	○	○	○	○	○	○	○
Are limited as to the products you can buy?	○	○	○	○	○	○	○
Must read labels?	○	○	○	○	○	○	○
Have the feeling that you have less control of what you eat when eating out?	○	○	○	○	○	○	○
Must refuse many things during social activities?	○	○	○	○	○	○	○
Sometimes frustrate people when they are making an effort to accommodate your food allergy?	○	○	○	○	○	○	○
Are less able to accept spontaneously an invitation to stay for a meal?	○	○	○	○	○	○	○

Are less able to taste or try various products when eating out?	○	○	○	○	○	○	○
Can eat out less?	○	○	○	○	○	○	○
Must personally check whether you can eat something when eating out?	○	○	○	○	○	○	○
Hesitate eating a product when you have doubts about it?	○	○	○	○	○	○	○

How troublesome is it, because of your shrimp allergy...

	Not	Barely	Slightly	Moderately	Quite	Very	Extremely
That the ingredients of a product change?	○	○	○	○	○	○	○
That labels are incomplete?	○	○	○	○	○	○	○
That the lettering on labels is too small?	○	○	○	○	○	○	○
That the label states: 'May contain (traces of shellfish)...'?	○	○	○	○	○	○	○
That ingredients are different in other countries (e.g. during vacation)?	○	○	○	○	○	○	○
That people underestimate your problems caused by shrimp allergy?	○	○	○	○	○	○	○
That is it unclear to which food you are allergic?	○	○	○	○	○	○	○
That you must explain to those around you that you have a shrimp allergy?	○	○	○	○	○	○	○
For your host or hostess should you have an allergic reaction?	○	○	○	○	○	○	○

How worried are you because of your shrimp allergy...

	Not	Barely	Slightly	Moderately	Quite	Very	Extremely
About your health?	○	○	○	○	○	○	○
That the allergic reactions to shrimp will become increasingly severe?	○	○	○	○	○	○	○

How frightened are you because of your shrimp allergy...

	Not	Barely	Slightly	Moderately	Quite	Very	Extremely
Of an allergic reaction?	◯	◯	◯	◯	◯	◯	◯
Of accidentally eating the wrong food?	◯	◯	◯	◯	◯	◯	◯
Of an allergic reaction when eating out despite the fact that your dietary restrictions have been discussed beforehand?	◯	◯	◯	◯	◯	◯	◯

Answer the following questions:

	Not	Barely	Slightly	Moderately	Quite	Very	Extremely
To what degree do you feel you are being a nuisance because you have a shrimp allergy when eating out?	◯	◯	◯	◯	◯	◯	◯
How discouraged do you feel during an allergic reaction?	◯	◯	◯	◯	◯	◯	◯
How apprehensive are you about eating something you have never eaten before?	◯	◯	◯	◯	◯	◯	◯

Food allergy independent measure - adult form (≥18 years)

How great do you think the chance is you...

	Never (0% chance)	Very small chance	Small chance	Fair Chance	Great Chance	Very Great Chance	Always (100% chance)
Will accidentally eat something to which you are allergic?	◯	◯	◯	◯	◯	◯	◯
Will have a severe reaction if you accidentally eat something to which you are allergic?	◯	◯	◯	◯	◯	◯	◯
Will die if you accidentally eat something to which you are allergic?	◯	◯	◯	◯	◯	◯	◯
Cannot effectively deal with an allergic reaction should you accidentally eat something to which you are allergic?	◯	◯	◯	◯	◯	◯	◯

How many products must you avoid because of your shrimp allergy?

◯ Almost none
◯ Very few
◯ A few
◯ Some
◯ Many
◯ Very many
◯ Almost all

How great is the impact of your shrimp allergy on your social life?

◯ Negligibly small
◯ Very small
◯ Small
◯ Moderate
◯ Great
◯ Very great
◯ Extremely great

How interested would you be in a treatment for shrimp allergy?

◯ Very interested
◯ Interested
◯ Neutral
◯ Not interested
◯ Extremely not interested

In response to having an interest in treatment, Do you want to be...
(Select all that apply)

You may also enter additional free text information in the next question.

☐ Protected from cross contamination (ie "bite proof" protection)
☐ Able to eat a serving size without reaction?

Why?

Do you have a shrimp allergic child (0 years to 17 years of age) that you would like to include in this survey?	◯ Yes<br>◯ No
Do you have a shrimp allergic child between the ages of 13 years old to 17 years old?	◯ Yes<br>◯ No

Shrimp Allergy Quality of Life Questionnaire Adolescent (age 13 years - 17 years)

The following questions concern the influence your shrimp allergy has on your quality of life. Answer every question by marking the appropriate box with an 'x'. You may choose from one of the following answers.

0= Not
1=Barely
2=Slightly
3=Moderately
4=Quite
5=Very
6=Extremely

How troublesome do you find it, because of your shrimp allergy, that you...

	Not	Barely	Slightly	Moderately	Quite	Very	Extremely
Must always be alert as to what you are eating?	○	○	○	○	○	○	○
Are able to eat fewer products?	○	○	○	○	○	○	○
Are limited as to the products you can buy?	○	○	○	○	○	○	○
Must read labels?	○	○	○	○	○	○	○
Have the feeling that you have less control of what you eat when eating out?	○	○	○	○	○	○	○
Must refuse treats at school or work?	○	○	○	○	○	○	○
Are less able to spontaneously accept an invitation to stay for a meal? Are less able to taste or try various products when eating out? Must check yourself whether you can eat something when eating out? Hesitate eating a product when you have doubts about it?	○	○	○	○	○	○	○
Must be careful about touching certain foods?	○	○	○	○	○	○	○
Must carry an epinephrine auto-injector?	○	○	○	○	○	○	○

Do you have an epinephrine auto-injector?
- ○ No
- ○ Yes

How troublesome is it, because of your shrimp allergy...

	Not	Barely	Slightly	Moderately	Quite	Very	Extremely
That the ingredients of a food change?	○	○	○	○	○	○	○
That the labeling of the bulk packaging (for example box or bag) is different than the individual packages?	○	○	○	○	○	○	○
That you have to explain to people around you that you have a food allergy?	○	○	○	○	○	○	○
That the label states: 'May contain (traces of shellfish)...'?	○	○	○	○	○	○	○

That during social activities others can eat shellfish ?	○	○	○	○	○	○	○
That during social activities your shrimp allergy is not taken into account enough?	○	○	○	○	○	○	○

How frightened are you because of your shrimp allergy...

	Not	Barely	Slightly	Moderately	Quite	Very	Extremely
Of an allergic reaction?	○	○	○	○	○	○	○
Of accidentally eating something wrong?	○	○	○	○	○	○	○
To eat something you have never eaten before?	○	○	○	○	○	○	○

Answer the following questions...

	Not	Barely	Slightly	Moderately	Quite	Very	Extremely
How discouraged do you feel during an allergic reaction?	○	○	○	○	○	○	○
How disappointed are you when people don't take your food allergy into account?	○	○	○	○	○	○	○

PART B:
The following four questions are about the chance that you think you have of something happening to you because of your shrimp allergy. Choose one of the answers. This is followed by two more questions about your shrimp allergy. Answer every question by putting an 'x' in the box next to the proper answer.

0=Never (0% chance)
1=Very small chance
2,=Small chance
3=Fair Chance
4=Great Chance
5=Very Great Chance
6=Always (100% chance)

How great do you think the chance is that you...

	Never (0% chance)	Very small chance	Small chance	Fair Chance	Great Chance	Very Great Chance	Always (100% chance)
Will accidentally eat something to which you are allergic?	○	○	○	○	○	○	○
Will have a severe reaction if you accidentally eat something to which you are allergic?	○	○	○	○	○	○	○

Will die if you accidentally eat something to which you are allergic?	◯	◯	◯	◯	◯	◯	◯
Can not effectively deal with an allergic reaction should you accidentally eat something to which you are allergic?	◯	◯	◯	◯	◯	◯	◯

How many products must you avoid because of your shrimp allergy?

◯ Almost none
◯ Very few
◯ A few
◯ Some
◯ Many
◯ Very many
◯ Almost all

How great is the impact of your shrimp allergy on your social life?

◯ Negligibly small
◯ Very small
◯ Small
◯ Moderate
◯ Great
◯ Very great
◯ Extremely great

How interested would you be in a treatment for shrimp allergy?

◯ Very interested
◯ Interested
◯ Not interested
◯ Extremely Not Interested

In response to having an interest in treatment, Do you want to be...
(Select all that apply)

You may also enter additional free text information in the next question.

☐ Protected from cross contamination (ie "bite proof" protection)
☐ Able to eat a serving size without reaction?

Why?

____

PART 1: MY CHILD'S FOOD ALLERGY

What sex are you?

◯ Male
◯ Female

What sex is your shrimp allergic child?

◯ Male
◯ Female

Q3. What age is the child with shrimp allergy?	◯ Less than one year<br>◯ 1 year<br>◯ 2 years<br>◯ 3 years<br>◯ 4 years<br>◯ 5 years<br>◯ 6 years<br>◯ 7 years<br>◯ 8 years<br>◯ 9 years<br>◯ 10 years<br>◯ 11 years<br>◯ 12 years<br>◯ 13 years<br>◯ 14 years<br>◯ 15 years<br>◯ 16 years<br>◯ 17 years
What type of food(s) is your child allergic to? Tick where applicable.	☐ Peanut<br>☐ Nut<br>☐ Milk<br>☐ Egg<br>☐ Wheat<br>☐ Soya<br>☐ Sesame<br>☐ Fish<br>☐ Shellfish<br>☐ Fruits<br>☐ Vegetables<br>☐ Other
Please specify 'Other':	__________________________________
Which of the above foods caused your child's most severe reaction?	◯ Peanut<br>◯ Nut<br>◯ Milk<br>◯ Egg<br>◯ Wheat<br>◯ Soya<br>◯ Sesame<br>◯ Fish<br>◯ Shellfish<br>◯ Fruits<br>◯ Vegetables<br>◯ Other
Please list which "other" food:	__________________________________
Has your child had an anaphylactic reaction?	◯ Yes<br>◯ No

To which food was the most recent reaction?	◯ Peanut<br>◯ Nut<br>◯ Milk<br>◯ Egg<br>◯ Wheat<br>◯ Soya<br>◯ Sesame<br>◯ Fish<br>◯ Shellfish<br>◯ Fruits<br>◯ Vegetables<br>◯ Other
Please list which food is "other":	__________________________________
If 'Yes', how recent was the reaction? Tick where applicable.	◯ Very recently<br>◯ 6 to 12 months ago<br>◯ Approximately 1 yrs ago<br>◯ Approximately 2 yrs ago<br>◯ More than 2 yrs ago
Has your child been issued with an epinephrine injector?	◯ Yes<br>◯ No

Does the provision of an epinephrine injector cause?

	For you	For your child
Reassurance...	☐	☐
Anxiety...	☐	☐

Who diagnosed your child with food allergy? Tick where applicable.	◯ Primary Care Provider<br>◯ Consultant Allergist<br>◯ Consultant Pediatrician<br>◯ Dermatologist<br>◯ Dietician<br>◯ Alternative Practitioner

What symptoms does your child have? Tick where applicable.

- ☐ Itching in the mouth
- ☐ Throat tightening
- ☐ Urticaria/Hives
- ☐ Itching in the throat
- ☐ Difficulty swallowing
- ☐ Skin swelling
- ☐ Itching in the ears
- ☐ Hoarseness
- ☐ Nausea
- ☐ Itching in the lips
- ☐ Difficulty breathing
- ☐ Abdominal cramps
- ☐ Runny nose
- ☐ Shortness of breath
- ☐ Vomiting
- ☐ Stuffy nose
- ☐ Wheeze
- ☐ Diarrhea
- ☐ Sneeze
- ☐ Cough
- ☐ Light-headedness
- ☐ Itchy eyes
- ☐ Itching of the skin
- ☐ Palpitations
- ☐ Tears
- ☐ Redness of the skin
- ☐ Inability to stand
- ☐ Red eyes
- ☐ Increase eczema
- ☐ Loss of consciousness

How often does your child meet another child with food allergy?

- ◯ Never
- ◯ Rarely
- ◯ Sometimes
- ◯ Often

PART 2: YOU AND YOUR CHILD'S WORRIES ABOUT FOOD SAFETY

Please answer the following questions with references to the 6-point scale.

0-Extremely unlikely
1-Very unlikely
2-Somewhat unlikely
3-Likely
4-Quite likely
5-Very likely
6-Extremely likely

What chance do you think your child has of...?

	Extremely unlikely	Very unlikely	Somewhat unlikely	Likely	Quite likely	Very likely	Extremely likely
...accidentally ingesting the food to which they are allergic?	◯	◯	◯	◯	◯	◯	◯

...having a severe reaction if food is accidentally ingested?	◯	◯	◯	◯	◯	◯	◯
...dying from his/her food allergy following ingestion in the future?	◯	◯	◯	◯	◯	◯	◯
...effectively treating him/herself, or receiving effective treatment from others (including Epipen administration), if he/she accidentally ingests a food to which he/she is allergic?	◯	◯	◯	◯	◯	◯	◯

What chance does your child think he/she has of...?

	Extremely unlikely	Very unlikely	Somewhat unlikely	Likely	Quite likely	Very likely	Extremely likely
...accidentally ingesting the food to which they are allergic?	◯	◯	◯	◯	◯	◯	◯
...having a severe reaction if food is accidentally ingested?	◯	◯	◯	◯	◯	◯	◯
...dying from his/her food allergy following ingestion in the future?	◯	◯	◯	◯	◯	◯	◯
...effectively treating him/herself, or receiving effective treatment from others (including Epipen administration), if he/she accidentally ingests a food to which he/she is allergic?	◯	◯	◯	◯	◯	◯	◯

What chance does your child think he/she has of...?

	Extremely unlikely	Very unlikely	Somewhat unlikely	Likely	Quite likely	Very likely	Extremely likely
...accidentally ingesting the food to which they are allergic?	◯	◯	◯	◯	◯	◯	◯
...having a severe reaction if food is accidentally ingested?	◯	◯	◯	◯	◯	◯	◯
...dying from his/her food allergy following ingestion in the future?	◯	◯	◯	◯	◯	◯	◯

...effectively treating him/herself, or receiving effective treatment from others (including Epipen administration), if he/she accidentally ingests a food to which he/she is allergic?	◯	◯	◯	◯	◯	◯	◯

How many foods does your child have to avoid?	◯ 0-2<br>◯ 3-6<br>◯ 7-10<br>◯ 10+
PART 3: YOUR CONCERNS AS A PARENT	
How would you describe...Your general health?	◯ Excellent<br>◯ Very good<br>◯ Fairly good<br>◯ Not so good<br>◯ Poor<br>◯ Very Poor
How would you describe...Your childs general health?	◯ Excellent<br>◯ Very good<br>◯ Fairly good<br>◯ Not so good<br>◯ Poor<br>◯ Very Poor
Because of shrimp allergy, how much worry/concern does each of the following cause you?<br><br>Your child's physical health?	◯ None at all<br>◯ A little bit<br>◯ Some<br>◯ Quite a bit<br>◯ A lot
Because of shrimp allergy, how much worry/concern does each of the following cause you?<br><br>Your child's emotional wellbeing?	◯ None at all<br>◯ A little bit<br>◯ Some<br>◯ Quite a bit<br>◯ A lot
What level of stress does your child's shrimp allergy cause... You?	◯ None at all<br>◯ A little bit<br>◯ Some<br>◯ Quite a bit<br>◯ A lot
What level of stress does your child's shrimp allergy cause...Your partner?	◯ None at all<br>◯ A little bit<br>◯ Some<br>◯ Quite a bit<br>◯ A lot

What level of stress does your child's shrimp allergy cause... Your family?

◯ None at all
◯ A little bit
◯ Some
◯ Quite a bit
◯ A lot

How much has shrimp allergy limited the type of activities... You can do as a family?

◯ None at all
◯ A little bit
◯ Some
◯ Quite a bit
◯ A lot

How much has shrimp allergy limited the type of activities...Your child can take part in?

◯ None at all
◯ A little bit
◯ Some
◯ Quite a bit
◯ A lot

How much has shrimp allergy limited the type of activities... Your child can take part in?

◯ None at all
◯ A little bit
◯ Some
◯ Quite a bit
◯ A lot

How interested would you be in a treatment for shrimp allergy for your child?

◯ Very interested
◯ Interested
◯ Neutral
◯ Not interested
◯ Extremely not interested

In response to having an interest in treatment, Do you want to be...
(Select all that apply)

You may also enter additional free text information in the next question.

☐ Protected from cross contamination (ie "bite proof" protection)
☐ Able to eat a serving size without reaction?

Why?

____

Shrimp Allergy Quality of Life Questionnaire - Children aged 0 - 12 years

Instructions to Parents

The following are scenarios that parents have told us affect children's quality of life because of shrimp allergy. Please indicated how much of an impact each scenario has on your child's quality of life by placing a tick or an x in one of the boxes numbered 0 - 6.

All information given is completely confidential. This questionnaire will only be identified by a code number.

There are 4 sections to this questionnaire: A, B, C, and D

If your child is aged 0 to 3 years, please answer section A

If your child is aged 4 to 6 years, please answer Section A and Section B

If your child is aged 7 years and over, please answer Section A, Section B, and Section C

Section D: For all age groups

0 = Not at all
1= A little bit
2= Slightly
3= Moderately
4= Quite a bit
5= Very much
6= Extremely

Do you have a shrimp allergic child who is 0-12 years old?	◯ Yes<br>◯ No

---

SECTION A:

If your child is aged 0 to 3 years, 4 to 6 years, or 7 years and over, please answer section A. (This refers to a child who is 0-12 years old)

Because of shrimp allergy, my child feels...

	Not at all	A little bit	Slightly	Moderately	Quite a bit	Very much	Extremely
Worried about food	○	○	○	○	○	○	○
Different from other children	○	○	○	○	○	○	○
Frustrated by dietary restrictions	○	○	○	○	○	○	○
Afraid to try unfamiliar foods	○	○	○	○	○	○	○
Concerned that I am worried that he/she will have a reaction to food	○	○	○	○	○	○	○

Because of shrimp allergy, my child...

	Not at all	A little bit	Slightly	Moderately	Quite a bit	Very much	Extremely
Experiences physical distress	◯	◯	◯	◯	◯	◯	◯
Experiences emotional distress	◯	◯	◯	◯	◯	◯	◯
Has a lack of variety in his/her diet	◯	◯	◯	◯	◯	◯	◯

Because of shrimp allergy, my child has been negatively affected by...

	Not at all	A little bit	Slightly	Moderately	Quite a bit	Very much	Extremely
Receiving more attention more attention than other children of his/her age	○	○	○	○	○	○	○
Having to grow up more quickly than other children of his/her age	○	○	○	○	○	○	○
His/her environment being more restricted than other children of his/her age	○	○	○	○	○	○	○

Because of shrimp allergy, my child's social environment is restricted because of limitations on...

	Not at all	A little bit	Slightly	Moderately	Quite a bit	Very much	Extremely
Restaurants we can safely go to as a family	○	○	○	○	○	○	○
Holiday destinations we can safely go to as a family	○	○	○	○	○	○	○

Because of shrimp allergy, my child's ability to take part has been limited...

	Not at all	A little bit	Slightly	Moderately	Quite a bit	Very much	Extremely
In social activities in other people's houses (sleepovers, parties, playtime)	◯	◯	◯	◯	◯	◯	◯

SECTION: B

if your child is aged 4 to 6 years, or 7 years and older, please answer Section A (previous section) and Section B

Is your shrimp allergic child between the ages of 4 years and 12 years old?

◯ Yes
◯ No

Because of shrimp allergy, my child's ability to take part has been limited...

	Not at all	A little bit	Slightly	Moderately	Quite a bit	Very much	Extremely
In preschool/school events involving food (class parties/treats/lunchtime)	○	○	○	○	○	○	○

Because of shrimp allergy, my child feels...

	Not at all	A little bit	Slightly	Moderately	Quite a bit	Very much	Extremely
Worried when going to unfamiliar places	○	○	○	○	○	○	○
Concerned that he/she must always be cautious about food	○	○	○	○	○	○	○
'Left out' in activities involving food	○	○	○	○	○	○	○
Upset that family social outings have been restricted by the need to plan ahead	○	○	○	○	○	○	○
Concerned about accidentally eating an ingredient to which he/she is allergic	○	○	○	○	○	○	○
Worried when eating with unfamiliar adults/children	○	○	○	○	○	○	○
frustrated by social restrictions	○	○	○	○	○	○	○

Because of shrimp allergy, my child...

	Not at all	A little bit	Slightly	Moderately	Quite a bit	Very much	Extremely
Is more worried in general than other children of his/her age	◯	◯	◯	◯	◯	◯	◯
Is more cautious in general than other children of his/her age	◯	◯	◯	◯	◯	◯	◯
Is not as confident as other children of his/her age in social situations	◯	◯	◯	◯	◯	◯	◯
Wishes his/her food allergy would go away	◯	◯	◯	◯	◯	◯	◯

SECTION: C

if your child is aged 7 years to 12 years old, please complete Section C.

Is your shrimp allergic child age 7 years to 12 years old?
◯ Yes
◯ No

SECTION: C if your child is aged 7 years and older, please answer Section A, Section B, and Section C

Because of shrimp allergy, my child...

	Not at all	A little bit	Slightly	Moderately	Quite a bit	Very much	Extremely
Worried about his/her future (opportunities, relationships)	◯	◯	◯	◯	◯	◯	◯
Many people do not understand the serious nature of food allergy	◯	◯	◯	◯	◯	◯	◯
Concerned by poor labeling on food products	◯	◯	◯	◯	◯	◯	◯
Food allergy limits his/her life in general	◯	◯	◯	◯	◯	◯	◯

Section D: For all age groups

Part 1: My child's shrimp allergy.

What sex are you?
◯ Male
◯ Female

What sex is your child?
◯ Male
◯ Female

What age is the child with shrimp allergy?	◯ Less than one year<br>◯ 1 year<br>◯ 2 years<br>◯ 3 years<br>◯ 4 years<br>◯ 5 years<br>◯ 6 years<br>◯ 7 years<br>◯ 8 years<br>◯ 9 years<br>◯ 10 years<br>◯ 11 years<br>◯ 12 years<br>◯ 13 years<br>◯ 14 years<br>◯ 15 years<br>◯ 16 years<br>◯ 17 years
What type of food(s) is your child allergic to? Tick where applicable	☐ Peanut<br>☐ Nut<br>☐ Milk<br>☐ Egg<br>☐ Wheat<br>☐ Soya<br>☐ Sesame<br>☐ Fish<br>☐ Shellfish<br>☐ Fruits<br>☐ Vegetables<br>☐ Other
Please specify 'Other'	__________________________________
Which of the above foods caused your child's most severe reaction?	☐ Peanut<br>☐ Nut<br>☐ Milk<br>☐ Egg<br>☐ Wheat<br>☐ Soya<br>☐ Sesame<br>☐ Fish<br>☐ Shellfish<br>☐ Fruits<br>☐ Vegetables<br>☐ Other
Please specify 'Other'	__________________________________
Has your child had an anaphylactic reaction?	◯ Yes<br>◯ No

To which food was the most recent reaction?	☐ Peanut<br>☐ Nut<br>☐ Milk<br>☐ Egg<br>☐ Wheat<br>☐ Soya<br>☐ Sesame<br>☐ Fish<br>☐ Shellfish<br>☐ Fruits<br>☐ Vegetables<br>☐ Other
Please specify 'Other'	______________________________
If 'Yes', how recent was the reaction? Tick where applicable.	◯ Very recently<br>◯ 6 to 12 months ago<br>◯ Approximately 1 yrs ago<br>◯ Approximately 2 yrs ago<br>◯ More than 2 yrs ago
Has your child been issued with an epinephrine auto-injector?	◯ Yes<br>◯ No
Does the provision of an epinephrine auto-injector cause? Reassurance?	☐ For you<br>☐ For your child
Does the provision of an epinephrine auto-injector cause? Anxiety?	☐ For you<br>☐ For your child
Who diagnosed your child with food allergy? Tick where applicable.	◯ Primary care provider<br>◯ Consultant Allergist<br>◯ Consultant Pediatrician<br>◯ Dermatologist<br>◯ Dietician<br>◯ Alternative Practitioner

What symptoms does your child have? Tick where applicable.

- ☐ Itching in the mouth
- ☐ Throat tightening
- ☐ Urticaria/Hives
- ☐ Itching in the throat
- ☐ Difficulty swallowing
- ☐ Skin swelling
- ☐ Itching in the ears
- ☐ Hoarseness
- ☐ Nausea
- ☐ Itching in the lips
- ☐ Difficulty breathing
- ☐ Abdominal cramps
- ☐ Runny nose
- ☐ Shortness of breath
- ☐ Vomiting
- ☐ Stuffy nose
- ☐ Wheeze
- ☐ Diarrhea
- ☐ Sneeze
- ☐ Cough
- ☐ Light-headedness
- ☐ Itchy eyes
- ☐ Itching of the skin
- ☐ Palpitations
- ☐ Tears
- ☐ Redness of the skin
- ☐ Inability to stand
- ☐ Red eyes
- ☐ Increase eczema
- ☐ Loss of consciousness

How often does your child meet another child with food allergy?

- ○ Never
- ○ Rarely
- ○ Sometimes
- ○ Often

Part 2: You and your child's worries about food safety.

Please answer the following questions with references to the 6-point scale.

0, Extremely unlikely
1, Very unlikely
2, Somewhat unlikely
3, Likely
4, Quite likely
5, Very likely
6, Extremely likely

What chance do you think your child has of...?

	Extremely unlikely	Very unlikely	Somewhat unlikely	Likely	Quite likely	Very likely	Extremely likely
...accidentally ingesting the food to which they are allergic?	○	○	○	○	○	○	○

...having a severe reaction if food is accidentally ingested?	◯	◯	◯	◯	◯	◯	◯
...dying from his/her food allergy following ingestion in the future?	◯	◯	◯	◯	◯	◯	◯
...effectively treating him/herself, or receiving effective treatment from others (including Epipen administration), if he/she accidentally ingests a food to which he/she is allergic?	◯	◯	◯	◯	◯	◯	◯

What chance does your child think he/she has of...?

	Extremely unlikely	Very unlikely	Somewhat unlikely	Likely	Quite likely	Very likely	Extremely likely
...accidentally ingesting the food to which they are allergic?	◯	◯	◯	◯	◯	◯	◯
...having a severe reaction if food is accidentally ingested?	◯	◯	◯	◯	◯	◯	◯
...dying from his/her food allergy following ingestion in the future?	◯	◯	◯	◯	◯	◯	◯
...effectively treating him/herself, or receiving effective treatment from others (including Epipen administration), if he/she accidentally ingests a food to which he/she is allergic?	◯	◯	◯	◯	◯	◯	◯

How many foods does your child have to avoid?

◯ 0-2
◯ 3-6
◯ 7-10
◯ 10+

How would you describe...

Your general health?

◯ Excellent
◯ Very good
◯ Fairly good
◯ Not so good
◯ Poor
◯ Very Poor

How would you describe...

Your child's general health?

◯ Excellent
◯ Very good
◯ Fairly good
◯ Not so good
◯ Poor
◯ Very Poor

Because of shrimp allergy, how much worry/concern does each of the following cause you?<br><br>Your child's physical health?	◯ None at all<br>◯ A little bit<br>◯ Some<br>◯ Quite a bit<br>◯ A lot
Because of shrimp allergy, how much worry/concern does each of the following cause you?<br><br>Your child's emotional well-being	◯ None at all<br>◯ A little bit<br>◯ Some<br>◯ Quite a bit<br>◯ A lot
What level of stress does your child's shrimp allergy cause...<br><br>You?	◯ None at all<br>◯ A little bit<br>◯ Some<br>◯ Quite a bit<br>◯ A lot
What level of stress does your child's shrimp allergy cause...<br><br>Your partner?	◯ None at all<br>◯ A little bit<br>◯ Some<br>◯ Quite a bit<br>◯ A lot
What level of stress does your child's shrimp allergy cause...<br><br>Your family?	◯ None at all<br>◯ A little bit<br>◯ Some<br>◯ Quite a bit<br>◯ A lot
How much has shrimp allergy limited the type of activities...<br><br>You can do as a family?	◯ None at all<br>◯ A little bit<br>◯ Some<br>◯ Quite a bit<br>◯ A lot
How much has shrimp allergy limited the type of activities...<br><br>Your child can take part in?	◯ None at all<br>◯ A little bit<br>◯ Some<br>◯ Quite a bit<br>◯ A lot
How interested would you be in a treatment for shrimp allergy for your child?	◯ Very interested<br>◯ Interested<br>◯ Neutral<br>◯ Not interested<br>◯ Extremely not interested

In response to having an interest in treatment, Do you want to be...
(Select all that apply)

You may also enter additional free text information in the next question.

☐ Protected from cross contamination (ie "bite proof" protection)
☐ Able to eat a serving size without reaction?

Why?
